# Study on Adjuvant Medication for Patients with Mild Cognitive Impairment Based on VR Technology and Health Education

**DOI:** 10.1155/2021/1187704

**Published:** 2021-12-07

**Authors:** Huiling Liu, Xiaona Yang, Xinkun Wang, Xiaoyu Yang, Xusheng Zhang, Qi Li

**Affiliations:** ^1^Department of Family Medicine, Second Provincial People's Hospital of Gansu, Lanzhou, Gansu 730000, China; ^2^Surgical Intensive Care Unit, Second Provincial People's Hospital of Gansu, Lanzhou, Gansu 730000, China; ^3^Neurosurgery, Second Provincial People's Hospital of Gansu, Lanzhou, Gansu 730000, China; ^4^General Surgery, Second Provincial People's Hospital of Gansu, Lanzhou, Gansu 730000, China; ^5^Academic Management Section, Second Provincial People's Hospital of Gansu, Lanzhou, Gansu 730000, China

## Abstract

In order to improve the efficiency of auxiliary medication for patients with mild cognitive impairment, this paper proposes a method based on VR technology and health education. Sixty elderly patients with COPD and MCI admitted to a hospital from January 2019 to February 2020 were randomly divided into a control group and study group, with 50 cases in each group. On the basis of conventional drug therapy, health education, and respiratory muscle training, patients in the control group received routine lung rehabilitation training, while patients in the study group received lung rehabilitation training using the BioMaster virtual scene interactive rehabilitation training system. Both groups continued training for 12 weeks. Lung function indexes, 6-minute walking distance, COPD assessment test (CAT) score, and Montreal Cognitive Function Assessment Scale (MoCA) score were compared between the 2 groups before training and 4, 8, and 12 weeks after training. The experimental results show that, in the study group, the percentage of FEV_1_ in the predicted value at 8 weeks after training, the percentage of FEV_1_ in the predicted value at 12 weeks after training, and FEV_1_/FVC were higher than those in the control group (*P* < 0.05). There was no significant difference in 6-minute walking distance, CAT score, and MoCA score between the two groups before training (*P* > 0.05). Twelve weeks after training, patients in the study group had a longer 6-minute walking distance, a lower CAT score, and a higher MoCA score than those in the control group (*P* < 0.05). It is proved that the application of virtual reality technology in lung rehabilitation training of elderly COPD patients with MCI is effective, which can effectively improve the lung function, cognitive function, and exercise tolerance of the patients and reduce the symptoms of dyspnea and the efficiency of medication.

## 1. Introduction

With the aging of the population, the number of dementia patients is increasing year by year, their cognitive ability and self-care ability are declining, and mental and behavioral symptoms are frequent, which brings a heavy burden to families and society. Mild cognitive impairment is the prophase symptom of dementia, and the annual conversion rate to dementia is 12%, while that of normal elderly people is only 1%∼2%. Intervention on dementia and its high-risk group, MCI, has attracted the attention of many scholars [1]. In recent years, due to the popularity of computers and the continuous development of software development technology, computer-aided cognitive rehabilitation technology has been gradually applied to the research fields of dementia and MCI. This technology can provide a simple, economical, effective, flexible, and diverse training platform according to the degree of cognitive impairment and interests of patients, so as to improve their memory, attention, and communication skills and meet their individual needs. At present, dementia intervention is mainly cognitive training and daily functional training, while MCI intervention is mostly cognitive training. There are few research on the application of computer-aided cognitive rehabilitation technology. Cognitive training software is widely used in the field of CACR, which can adjust the difficulty of training according to the cognitive level and give answers and help to patients when they cannot complete the task. At present, it is mostly used for MCI patients, but the rehabilitation of dementia patients is relatively less [2]. The most commonly used one is neuropsychological training software, and this software was originally used for rehabilitation of aphasia patients and was introduced into the MCI and dementia intervention field. It can provide training according to patients' attention, memory, understanding ability, positioning ability, and language ability. Intelligent voice and captain's log software provides training in color or shape matching, digital calculation, audio-visual material recognition, etc. to improve the memory and attention of dementia patients [3]. Brain-strengthening software mainly improves the processing speed and accuracy of information such as visual space of MCI patients. Although the training emphasis provided by the abovementioned software is different, it has got rid of the traditional rehabilitation mode focusing on memory training. It mainly provides targeted, flexible, and adjustable multidirectional intervention activities according to different cognitive impairment areas and severity of patients, shortens intervention time, and gives timely feedback and objective evaluation to patients' executive ability [4].

In the 21st century, with the continuous development of computer and software development technology and the extensive use of the Internet, computer-aided cognitive rehabilitation technology is widely used in dementia diagnosis, course tracking, and caregiver support. For convenience of operation, some researchers use touch screen instead of mouse and keyboard and set up sound prompt [5]. Wen et al. first applied this technology to the intervention of dementia patients, providing human-computer interaction daily homework training such as doing housework and shopping based on the living environment or life-related photos. Despite the lack of rigor in the study design, these studies have expanded the field of nonpharmacological interventions for dementia [6]. Haussmann et al. used the software “MultiTask” to train patients with dementia, providing the practice of finding specified items and rooms in the set apartment scene, so as to improve the immediate memory and delayed memory related to images and orientation [7]. Guo et al. added sound hints on the basis of text guidance to ensure that illiterate or poor vision could operate. In terms of intervention, it is not limited to only the training of memory and daily living ability but also focuses on the improvement of attention, reasoning ability, communication, and social activity ability [8]. In the 21st century, CACR is developing towards diversification, individualization, and individuation in the research field of dementia, and the intervention objects are also expanded from dementia to MCI patients. The purpose of this study is to analyze the application effect of virtual reality technology in lung rehabilitation training for elderly COPD patients with MCI. To provide new ideas for improving the effect of pulmonary rehabilitation training, 60 elderly COPD patients with MCI admitted to a hospital from January 2019 to February 2020 were randomly divided into a control group and study group, with 50 cases in each group. The observation group adopted the intervention program of health promotion combined with health education under the guidance of community doctors, while the control group only received basic health education. Before and 12 weeks after the intervention, the Montreal Cognitive Assessment Scale (MoCA) was used to evaluate cognitive function, and ADL was used to evaluate activities of daily living.

## 2. Materials and Methods

### 2.1. General Information

Sixty elderly patients with COPD and MCI admitted to a hospital from January 2019 to February 2020 were randomly divided into a control group and study group, with 50 cases in each group. There were 38 males and 12 females in the control group. The average age was 74.6 years for males and 75.3 years for females Grade of COPD severity: 33 cases were moderate, and 17 cases were severe. There were 40 males and 10 females in the study group. The average age was 73.6 years for males and 76.3 years for females Grade of COPD severity: 30 cases were moderate, and 20 cases were severe. Comparing the general data between the two groups, there was no significant difference (*P* > 0.05), but it was comparable [9]. The study was approved by the hospital ethics committee, and all patients were informed of the study and signed informed consent.

### 2.2. Methods

After admission, the patients in both groups were treated with conventional drugs such as sputum removal, bronchiectasis, and inhaled glucocorticoid and were given health education and respiratory muscle training. The health education included explaining COPD-related knowledge, emphasizing the significance of lung rehabilitation training, and guiding patients to quit smoking and reasonably ingesting nutrients. The respiratory training method is as follows: the patient takes an upright sitting position or a supine position, relaxes the whole body, puts one hand flat on the abdomen and the other hand on the chest, closes the lips tightly, inhales slowly with the nose so that the hand placed on the abdomen rises with the inhalation of the abdomen, and then, exhales slowly with the whistle-mouth-shaped lips, so that the hand placed on the abdomen sinks with the exhalation. Attention is paid to that the hand placed on the chest remaining motionless during the whole inhalation and exhalation process, 30 min/time, once/d, 5 times/week, with continuous training for 12 weeks. (1) On the basis of the abovementioned treatment, patients in the control group received conventional lung rehabilitation training: upper limb training was carried out by arm lifting, side lifting, and circling, and lower limb training was carried out by walking, once a day. The initial training time was 5 min/time and gradually increased to 15 min/time. (2) Patients in the study group used the Bio Master virtual scene interactive rehabilitation training system for lung rehabilitation training and selected cycling simulation for lower limb training, once per day. The initial training time was 5 min/time and then gradually extended to 15 min/time according to the patient's training tolerance. Patients in both groups continued training for 12 weeks [10].

### 2.3. Observation Indicators

#### 2.3.1. Lung Function Index

The lung function indexes of patients in the two groups before training and at the 4th, 8th, and 12th weeks after training were measured with a lung function meter, and the percentage of FEV_1_ in the predicted value and the ratio of forced expiratory volume to forced vital capacity in the first second (FEV_1_/FVC) [11] were calculated.

#### 2.3.2. 6-minute Walking Distance

Patients in the two groups were given a 6-minute walking test before and 12 weeks after training: signs such as starting point, ending point, and turning direction were marked in a straight, hard corridor, or corridor with a length exceeding 25 m indoors, eye-catching signs were marked every 3 m, patients were instructed to walk at their maximum speed and given standard encouraging words, and the 6-minute walking distance of patients was measured after the test [12].

#### 2.3.3. Evaluation Test Score of COPD

CAT was used to evaluate the severity of dyspnea symptoms before and 12 weeks after training in two groups, including chest tightness, expectoration, cough, energy, sleep, emotion, exercise ability, and their influence on daily life, with each score being 0–5 points.

#### 2.3.4. MoCA Evaluation Test Score

The total score is 0∼40 points; the higher the CAT score, the more severe the symptoms of dyspnea. MoCA score used MoCA to evaluate the cognitive function of patients in the two groups before and 12 weeks after training, including attention, concentration, executive ability, language, memory, and calculation ability, with a full score of 30. The lower the score, the worse the cognitive function.

### 2.4. Statistical Methods

SPSS19.0 software was used for data analysis, and the measurement data were expressed in (*x s*). Two independent-sample *T*-tests were used for comparison between the two groups, and a paired *T*-test was used for comparison within the group. Repeated measurement data analysis adopts two-factor repeated measurement variance analysis; the ○^2^test was used for counting data analysis. *P* < 0.05 was statistically significant [13].

## 3. Results

### 3.1. Lung Function Indicators

**Figure 1:**
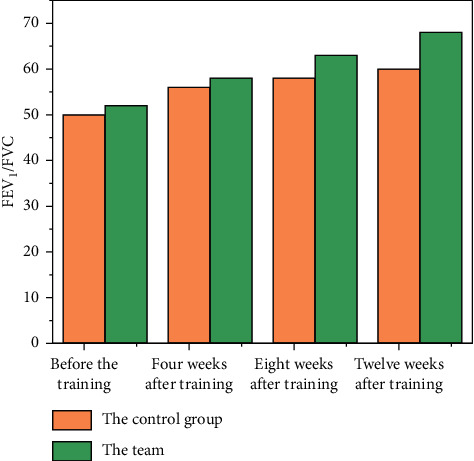
Comparison of FEV_1_ between two groups before training and 4, 8, and 12 weeks after training.

There is an interaction between time and method in the percentage of FEV_1_ to the predicted value and FEV_1_/FVC (*P* < 0.05). Also, the main effects of time and method on the percentage of FEV_1_to the predicted value and FEV_1_/FVC were significant (*P* < 0.05). The control group had an improvement, but the improvement was not obvious, while the research group had a significant increase. The percentage of FEV_1_ in 8 weeks after training, the percentage of FEV_1_ in 12 weeks after training, and FEV_1_/FVC in the study group were higher than those in the control group, and the difference was statistically significant (*P* < 0.05, see Table 1 and Figure 1) [14].

### 3.2. 6-Minute Walking Distance, CAT Score, and MoCA Score

**Figure 2:**
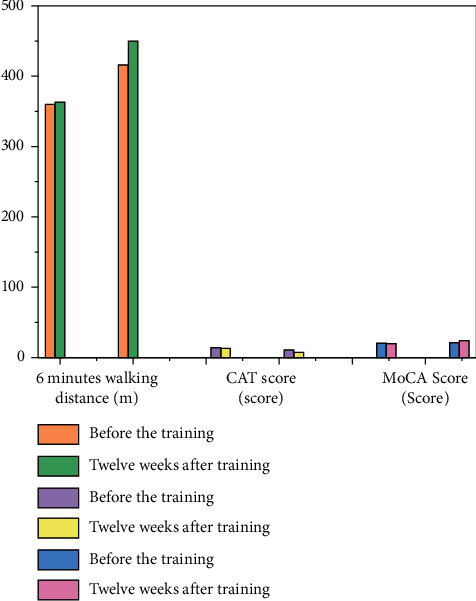
Comparison of walking distance, CAT score, and MoCA score between the two groups before and after training for 12 weeks and 6 minutes.

Before training, there was no significant difference in walking distance, CAT score, and MoCA score between the two groups (*P* > 0.05). Twelve weeks after training, the 6-minute walking distance, CAT score, and MoCA score of patients in the study group were longer than those in the control group, and the differences were statistically significant (*P* < 0.05, see Figure 2).

### 3.3. Comparison of MoCA Scores between the Two Groups before and after Intervention

Before intervention, there was no significant difference in MoCA scores and cognitive domain scores between the two groups (*P* > 0.05) [15]. At the 12th week after the intervention, the scores of visual space, executive ability, and attention of the patients in the observation group were significantly higher than those in the control group (*P* < 0.05). The 12th week after intervention, the scores of visual space, executive ability, naming, and attention of patients in the observation group were significantly higher than those before intervention, and the difference was statistically significant (*P* < 0.05).

### 3.4. Comparison of Activities of Daily Living between the Two Groups before and after Intervention

At the 12th week after the intervention, the ADL score of the observation group was significantly lower than that before the intervention, and the difference was statistically significant (*P* < 0.05). In the 12th week after the intervention, the ADL scores of the observation group were significantly lower than those of the control group, and the difference was statistically significant (*P* < 0.05) (see Table 2). Although CACR has a short history of rehabilitation training for PATIENTS with MCI and dementia, it has certain effects on improving patients' cognition, emotion, and communication advantages, but there are still some shortcomings.

## 4. Discussion

OPD is one of the common chronic respiratory diseases in clinic, with high morbidity, disability, and death rate, ranking the fourth cause of death of residents worldwide due to illness, and it is one of the world public health problems. According to statistics, more than 400,000 patients die from COPD and its complications every year [9].

At present, sports training is still the core content of lung rehabilitation training. On the one hand, patients need good cooperation and tolerance; on the other hand, patients need good execution, attention, and learning ability to ensure the effectiveness of sports training. However, elderly COPD patients are easily affected by factors such as age, educational level, and family support, which leads to poor compliance in sports training. In addition, the monotonicity of sports training also makes the elderly COPD patients' long-term persistent interest low, and the cognitive function is reduced or MCI affects them, resulting in poor sports training effect of the elderly COPD patients [16]. The incidence of MCI in COPD patients is about 10.4%. The incidence of MCI in stable COPD patients is about 8.8%, and that in acute exacerbation COPD patients is about 22.6%. Other research results show that only 3% of COPD patients have normal cognitive function. The intervention was carried out through well-designed training and activity programmes that relieved some of the work of dementia caregivers and reduced work stress. Therefore, how to ensure and maintain the effect of sports training for elderly patients with COPD and MCI has become one of the difficult problems in lung rehabilitation training. At present, there are more and more mature pulmonary rehabilitation training programs for elderly COPD patients. However, the lung rehabilitation training program for elderly COPD patients with MCI is still lack of pertinence. Clinical intervention is mainly carried out by actively treating primary diseases, preventing complications, and controlling disease progression. Studies have shown that ltot can effectively improve the cognitive function of elderly patients with COPD, but other studies have shown that ltot has limited effect on the cognitive function of elderly patients with COPD. Therefore, it is still necessary to actively explore a targeted lung rehabilitation training program [17] for elderly COPD patients with MCI.

At present, virtual reality technology has been gradually applied in many medical fields and has shown great advantages in the field of rehabilitation medicine, but most of it focuses on limb function rehabilitation after stroke and spinal cord injury, and there are few reports on its application in pulmonary rehabilitation training for COPD patients. The results of this study show that the FEV_1_/FVC ratio in the study group was higher than that in the control group 8 weeks after training and 12 weeks after training, while the 6-minute walking distance, CAT score, and MoCA score in the study group were longer than those in the control group 12 weeks after training, which indicated that virtual reality technology had a good effect in lung rehabilitation training for elderly COPD patients with MCI. It can effectively improve the lung function, cognitive function, and exercise tolerance of patients and alleviate the symptoms of dyspnea. The reasons mainly include the following two aspects: (1) virtual reality technology has increased the interest of lung rehabilitation training, and the operation difficulty and virtual scene can be adjusted individually, which is conducive to mobilizing the enthusiasm and initiative of elderly COPD and MCI patients to adhere to lung rehabilitation training; (2) virtual reality technology can mobilize patients' thinking, memory, and attention at the same time, which is beneficial to relieve patients' tension and fatigue and improve their cognitive function, thus achieving compliance with drug treatment [18].

The research shows that, with the development of a community intervention model for diseases, the cognitive function of patients with mild cognitive impairment can be improved after proper intervention. In this study, health education was combined with a health promotion program, which made patients have a more objective understanding of mild cognitive impairment after stroke, and cooperated with the three-dimensional intervention of characteristic health promotion measures such as acupoint massage, healthcare techniques, and foot bath with traditional Chinese medicine. The principle of TCM diagnosis and treatment of holistic thinking was followed to improve patients' cognitive function and behavioral ability. Through the communication between patients and the external environment, they stimulate their thinking and exercise their thinking ability. Meanwhile, TCM healthcare techniques and acupoint massage operations also strengthen the cyclic reflection process of patients' memory, cognition, and self-experience, promote the active activities of the brain, and improve their cognitive function. Traditional Chinese medicine foot massage, healthcare techniques, and acupoint massage directly strengthen the physical function exercise of patients and improve their daily behavior ability. Virtual reality technology increases the interest of pulmonary rehabilitation training, and the difficulty of operation and the personalized adjustment of virtual scenes are conducive to mobilizing the enthusiasm and initiative of elderly patients with COPD and MCI to adhere to pulmonary rehabilitation training; virtual reality technology can mobilize patients' thinking, memory, and attention at the same time, which is beneficial to relieve patients' tension and fatigue and improve their cognitive function; virtual reality program training can improve patients' participation enthusiasm and activity ability, but its conclusion lacks objective quantitative indicators to judge [19].

## 5. Conclusions

This paper proposes a virtual reality method which can effectively solve the problem of oral medication compliance of patients with mild cognitive impairment. The specific content of this method is to select suitable subjects for insight assessment, treatment compliance assessment, and cognitive dysfunction assessment, enhance autonomy, and improve daily activities. After the intervention, the scores of daily activity ability of patients in the experimental observation group were higher than those before the intervention and the control group (*P* < 0.05), which indicated that virtual reality technology could improve the daily activities ability of patients. To prove the effect of virtual reality in solving the problem of oral medication compliance of patients with mild cognitive impairment, it is suggested that immersive virtual reality technology can improve the psychological status of senile dementia patients.

## Figures and Tables

**Table 1:** Comparison of pulmonary function indexes before training and 4, 8, and 12 weeks after training between the two groups.

Group	Number of cases	The percentage of FEV_1_in the predicted value			
		Before training.	Four weeks after training.	Eight weeks after training.	Twelve weeks after training.
Control group	50	40.3 ± 10.7	45.8 ± 12.7	44.8 ± 5.9	51.7 ± 13.2
Research group	50	39.2 ± 8.6	44.0 ± 10.3	55.1 ± 8.3	57.7 ± 10.3
*P* value		*P* < 0.01	*P* < 0.01	*P* < 0.01	*P* < 0.01

**Table 2:** Comparison of ADL scores of patients before and after intervention.

Group	Before intervention.	After 12 weeks of intervention,	*P* value
Control group	28.5	22.13	0.026
Research group	28.17	25.9	0.125
Compared with the control group after intervention, *P* < 0.05			

## Data Availability

The data used to support the findings of this study are available from the corresponding author upon request.
